# Nursing Process Related to the Nursing Focus “Airway Clearance”: A Scoping Review

**DOI:** 10.3390/nursrep14030140

**Published:** 2024-07-31

**Authors:** Luís Gaspar, Neuza Reis, Paula Sousa, Abel Paiva e Silva, Alexandrina Cardoso, Alice Brito, Fernanda Bastos, Joana Campos, Paulo Parente, Filipe Pereira, Natália Machado

**Affiliations:** 1 Faculty of Health Sciences and Nursing, Universidade Universidade Católica Portuguesa, 4169-005 Porto, Portugal; neuza.reis@gmail.com; 2 Porto School of Nursing, Escola Superior de Enfermagem do Porto, 4200-072 Porto, Portugal; paula.sousa@esenf.pt (P.S.); abel@esenf.pt (A.P.e.S.); alex@esenf.pt (A.C.); alice@esenf.pt (A.B.); fbastos@esenf.pt (F.B.); joana@esenf.pt (J.C.); paulo@esenf.pt (P.P.); filipereira@esenf.pt (F.P.); natalia@esenf.pt (N.M.)

**Keywords:** advanced practice nursing, airway clearance, nursing, nursing process, scoping review

## Abstract

Background: Airway clearance impairment has a significant impact on self-care and quality of life. Identifying clinical data, nursing diagnoses, and nursing interventions is essential to clinical reasoning and enhancing nursing care. This study aims to map the existing evidence on clinical data, nursing diagnoses, and nursing interventions addressing the nursing focus on “airway clearance”. Methods: Research was conducted based on Joanna Briggs’s Scoping Review Methodology. We searched four databases for published studies until December 2023. Results: From the initial 1854 studies identified, 123 were included in the review. The findings highlighted two areas of nursing attention: one related to signs and symptom management, and the other related to education and coping strategies. The data that led to nursing diagnoses were divided into cognitive and clinical data. The nursing diagnoses were mostly related to secretion retention, excessive mucus production, and airway obstruction. The most commonly identified nursing interventions were educational interventions assembled into predesigned education programs rather than patient-tailored programs. Conclusions: Findings can add substantial value for systematizing the nursing process related to “airway clearance”, improving nursing decision-making and care quality. This study was prospectively registered with the Open Science Framework (OSF) on 02 December 2022, with the registration number wx5ze.

## 1. Introduction

The International Classification of Nursing Practice (ICNP) defines airway clearance as the process of “keeping air passage open from mouth to lung alveoli through the ability to clear secretions or obstruction from the respiratory tract” [1].

The mucociliary clearance and cough reflex protect the respiratory system by enabling pulmonary secretion clearance and preventing airway obstruction and infections.

Many factors can impair ciliary function, alter secretion production and mucus rheology, and interfere with the cough reflex. Aging, tobacco use, and environmental exposures reduce the efficacy of the ciliary structure [2]. Progressive neurodegenerative diseases like amyotrophic lateral sclerosis decrease and, in the majority of cases, inhibit cough [3]. Pulmonary conditions like COPD, bronchiectasis, or cystic fibrosis change the production and characteristics of the mucus, and mucociliary clearance disorders, such as primary ciliary dyskinesia, reduce the efficacy of ciliary structure and function [4]. Also, complications related to acute pulmonary infections, invasive ventilation-related problems, and thoracic and abdominal surgery make it difficult to mobilize and expel lung secretions.

There is a wide range of treatments, techniques, and devices for managing bronchial burden. Looking at this issue from the patient’s perspective, there is rarely just one single technique used for a given pathological condition. In addition, for many patients and/or categories of patients, the goal might be to combine the best effect with the lowest possible incidence of side effects and adverse events.

Nurses play an essential role in caring for patients with airway clearance impairment, whether by enhancing airway clearance or by stabilizing the impairment itself. The increased complexity of health problems and the need for patient-centered care required developing, improving, and mastering nurses’ intellectual, interpersonal, and technical abilities to make clinical decisions compatible with safe and effective practices [5]. This path leads to data standardization by introducing taxonomies, such as the ICNP or NANDA International Inc., allowing the use of standard language regarding nursing care and enhancing the continuity of care and nursing outcome production [6].

Without prejudice to the above, we set out to carry out the scoping review without a pre-defined and predetermined theoretical framework to guarantee maximum potential for the inclusion of results. However, considering the purpose of this review, we took into account the phases of the clinical nursing decision-making process [7].

The nursing process is a critical thinking model used to promote competent care. It includes five steps: assessment, diagnosis, planning, implementation, and evaluation [8]. In the assessment phase, nurses collect all the data essential for predicting, detecting, preventing, and managing actual and potential problems. In the diagnostic phase, nurses analyze the gathered data, draw conclusions, and determine whether there are real or potential problems requiring nursing management. In the third and fourth phases, nurses determine nursing interventions and implement the action plan [8]. Each phase of the nursing process should be conducted using a standardized nursing language since common terminology is crucial to present nursing practices [1]. Although airway clearance is a prevalent phenomenon in all contexts of care and is represented in all nursing classifications, no published studies have evaluated the nursing process related to this topic. Furthermore, despite some research evaluating the efficacy of nursing interventions, studies summarizing those interventions still need to be identified. This lack of research also extends to clinical data and nursing diagnoses, leading to inadequate knowledge.

Clinical reasoning, as well as clinical judgment or decision-making processes, are terms that commonly refer to the processes through which nurses guide their clinical practice. Nurses collect, analyze, and interpret daily airway clearance clinical-related data. This allows the creation of nursing diagnoses and, subsequently, the prescription, implementation, and evaluation of nursing interventions. Hence, nurses must identify and define the relationships among different datasets, transforming them into information that simultaneously includes the best scientific evidence and is applicable to ensuring better nursing care [9].

Currently, how nurses conceptualize and integrate airway clearance impairment into the nursing process is very heterogeneous. This fact creates a massive amount of different clinical data, limiting the exchange of information and the continuity of care and significantly impacting the best evidence-based delivery of nursing knowledge.

The evolution of nursing informatics has led to complex data processing systems that can help clinical reasoning, suggest solutions, and present outcomes of nursing care [10]. The development of nursing clinical data models provides evidence-based data, allowing information to be structured. This structuration will systematize the relations between the nursing process elements, describing complex information structures that indicate how information should be expressed and what is optional or mandatory according to current scientific evidence [11]. Combining clinical data, nursing diagnoses, and nursing interventions into a nursing-led classification will help translate the existing knowledge into precise, interoperable data, enhancing nursing care and providing a solid basis for nurses’ decision-making processes.

By resuming each step of the nursing process, this review can lead to a clinical data model comprising the elements of the nursing process centered on airway clearance.

We performed a preliminary search in PROSPERO, MEDLINE, the Cochrane Database of Systematic Reviews, and the Joanna Briggs Evidence Synthesis. No current or ongoing scoping or systematic reviews on the topic were identified.

This review aimed to map the evidence on clinical data, nursing diagnoses, and nursing interventions addressing the nursing focus on “airway clearance”. Thus, we focused on the following questions: (1) What clinical data do nurses use to identify nursing diagnoses related to the nursing focus “airway clearance”? (2) What nursing diagnoses are related to the nursing focus “airway clearance”? (3) What nursing interventions positively address nursing diagnoses focused on airway clearance?

## 2. Materials and Methods

This review was conducted according to the methodology of the Joanna Briggs Institute for scoping reviews [12]. In addition, we followed the Preferred Reporting Items for Systematic Reviews and Meta-Analyses Extension for Scoping Reviews (PRISMA-ScR) checklist [13]. The review protocol is registered in the Open Science Framework at https://doi.org/10.17605/OSF.IO/KN27G (accessed on 25 July 2024).

### 2.1. Search Methods

A comprehensive search strategy aimed to identify published studies related to “*airway clearance*”. The study identification used a three-step search approach [14]. The first step involved an initial limited search in the MEDLINE (PubMed) and CINAHL (EBSCO) databases to identify studies on the topic and then analyze the index terms and text words from their titles and abstracts. The second step was an extensive search (carried out on 19 March 2024), including all identified index terms and keywords across the following databases: CINAHL Complete, MedicLatina, MEDLINE (PubMed), and PEDro. In the third step, the reference lists of all the selected studies were screened for additional studies (see Table S1 in the Supplementary Materials).

### 2.2. Inclusion and Exclusion Criteria

The eligibility criteria for the studies were defined based on the PCC mnemonic (participants, concept, context) in line with the methodology proposed by the JBI [14].

#### 2.2.1. Participants

This scoping review considered all studies that included adult patients (above 18 years old) related to THE nursing focus “airway clearance”, except those linked to the deglutition process and those that included caregivers or parents.

#### 2.2.2. Concept

The concept of this scoping review is the nursing knowledge used in the nursing clinical reasoning process, particularly nursing assessment, nursing diagnosis, planning, and implementation of nursing interventions.

#### 2.2.3. Context

This scoping review included all studies developed in hospitals, primary care, and home care, regardless of country of origin or sociocultural setting.

#### 2.2.4. Types of Evidence Sources

This study considered quantitative, qualitative, and mixed-methods study designs. The quantitative designs included experimental, quasi-experimental study designs (including randomized controlled trials, non-randomized controlled trials, and other quasi-experimental studies) and observational designs (descriptive, cohort, cross-sectional, case, and case series studies). Qualitative designs include studies that focus on qualitative data, including phenomenology, grounded theory, and ethnographic designs. In addition, it included systematic reviews, texts, and opinion papers. Conference abstracts and posters were excluded from this review due to brevity. Only studies published in English, Spanish, and Portuguese until 31 December 2023 were considered.

#### 2.2.5. Study Selection

After the search, all identified records were uploaded into EndNote 8.0 (Clarivate Analytics, Philadelphia, PA, USA).

Two researchers (LG and NR) independently performed the study selection, and disagreements between reviewers were resolved through discussion with a third reviewer (NM).

### 2.3. Data Extraction and Analysis

Data were extracted from the included studies by LG and NR using a data extraction tool developed by the reviewers that were aligned with the objectives and research questions.

This tool was based on the Joanna Briggs model instrument for extracting details, characteristics, and results of the studies [14]. It included author[s], year of publication, study design, nursing diagnoses, clinical data, and interventions addressing the nursing focus on airway clearance.

Any disagreements were resolved through discussion with a third reviewer (NM). The two reviewers charted the first “ten studies using the data charting form and met to determine whether their approach to data extraction is consistent with the research question and purpose”, as Levac, Colquhoun, and O’Brien suggested [15].

#### Quality Appraisal

According to the scoping review methodology, the quality of the studies included in this review was not assessed.

## 3. Results

The review identified a total of 1854 studies. After removing the duplicates, 1750 articles were reviewed after considering the inclusion criteria by reading the title and abstract. Finally, 123 articles were considered for inclusion in the final dataset after full-text reading and analysis guided by the research questions.

**Figure 1:**
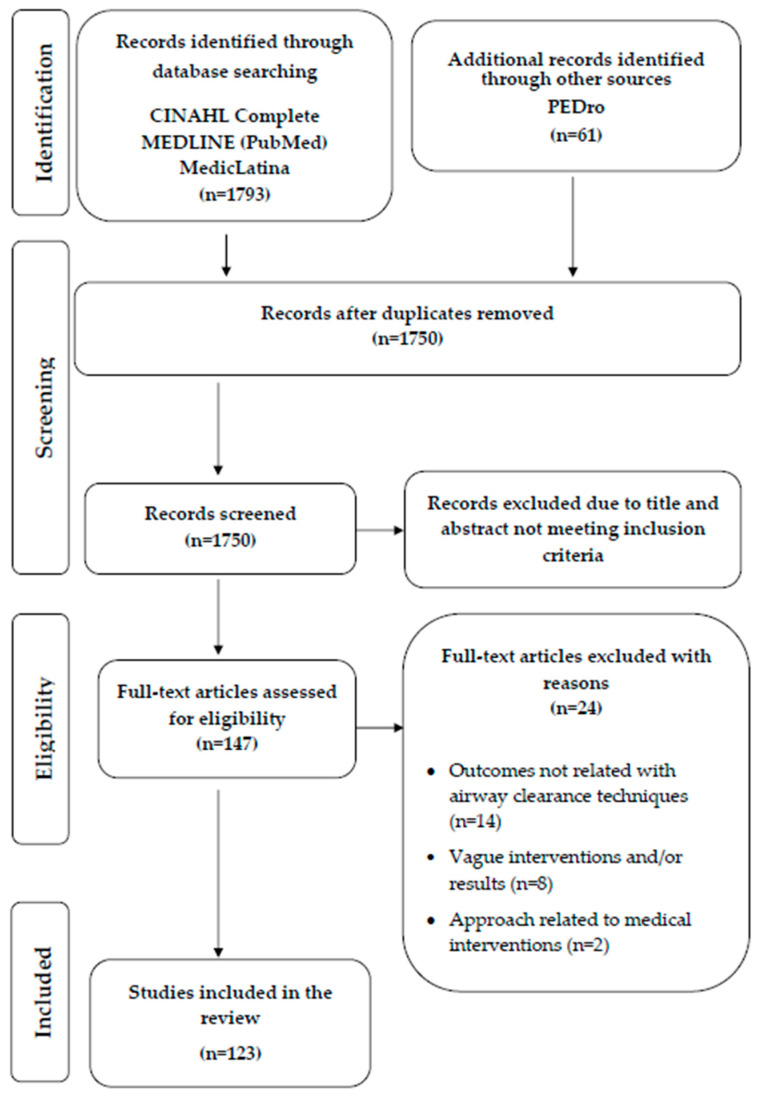
PRISMA flowchart.

The procedure used to select the included papers is presented in a flow diagram of the Preferred Reporting Items for Systematic Reviews and Meta-analyses (PRISMA) guidelines (Figure 1).

### 3.1. Study Characteristics

A total of 123 studies were included in this scoping review. Concerning the type of studies identified, the analysis included a combination of literature reviews and quantitative and mixed methods studies, with quantitative designs accounting for 63.4% of the total number of studies analyzed. Clinical studies were mainly performed in the United States of America (21.4%), the United Kingdom (18.7%), and Brazil (10.5%). The theme most frequently identified in the papers was “Interventions” (n = 258), followed by “Clinical Data” (n = 253), and “Diagnoses” (n = 159). The records included in this review are presented in Table 1, showing the clinical data, nursing diagnoses, and nursing interventions included in the studies.

The extracted data were evaluated to answer the research questions and underwent a basic descriptive analysis in accordance with JBI recommendations [14].

#### 3.1.1. Clinical Data

In this review, we encountered clinical data on clinical signs and symptoms and clinical data related to education strategies.

Regarding the data on clinical signs and symptoms, it reflected the physiological impact of impaired airway clearance on respiratory function (e.g., oxygen saturation (14.6%) or respiratory rate (2.8%)), the presence of bronchial secretions (e.g., breathing sounds (8.3%)), sputum amount (13%), and sputum characteristics (1.6%) (e.g., color, consistency, or odor). In addition, it was also found other clinical data that, despite not being directly linked to airway clearance, can also lead to impaired airway clearance, like data related to cough mechanisms (e.g., cough efficacy (10.3%), peak cough flow (7.9%), or cough reflex (4%)).

Concerning education strategies, we found data related to adherence to airway clearance (4.3%), knowledge (5.9%), and ability (6.7%) to perform airway clearance techniques, meaning about airway clearance techniques (2.4%), and awareness of airway clearance techniques (0.8%).

These results allow us to answer the research question (1), “What clinical data do nurses use to identify nursing diagnoses related to the nursing focus “airway clearance”?

#### 3.1.2. Nursing Diagnoses

Concerning the nursing diagnoses, the results were linked to secretion retention, excessive mucus production, and airway obstruction. Impaired airway clearance was the most common diagnosis found in the review (66.7%).

It was also found that some diagnoses comprise two nursing focuses, which is relevant for the nursing process addressing the focus “airway clearance”. Diagnoses such as “Lack of airway clearance knowledge” (11.3%) or “Lack of airway clearance adherence” (8.2%) were also mentioned and considered in this review.

These results answer the research question (2), “What nursing diagnoses are related to the nursing focus “airway clearance”?

#### 3.1.3. Nursing Interventions

Finally, regarding nursing interventions, the following results were related to research question 3, “What nursing interventions positively address nursing diagnoses focused on airway clearance”? The results found were: educational interventions (28.7%), manual airway clearance techniques (27.9%) (e.g., vibration maneuvers, active cycle of breathing, forced expiratory technique, and autogenic drainage), airway clearance with respiratory devices (22.5%) (e.g., oscillatory devices, mechanical insufflation/exsufflation devices, high-frequency chest wall compression devices), airway suction (8.5%), postural drainage (8.1%), promoting care plans (1.6%), and inspiratory muscle training (1.5%).

Considering that there are widely accepted international classifications of nursing diagnoses and interventions, such as NANDA-I, NIC, and ICNP, we decided to map the results obtained with those three classifications.

This semantic mapping process was rigorously conducted, adhering to the principles of the 2016 ISO 12300 standard and following the terms described by Torres et al. (2020) [137].

**Figure 2:**
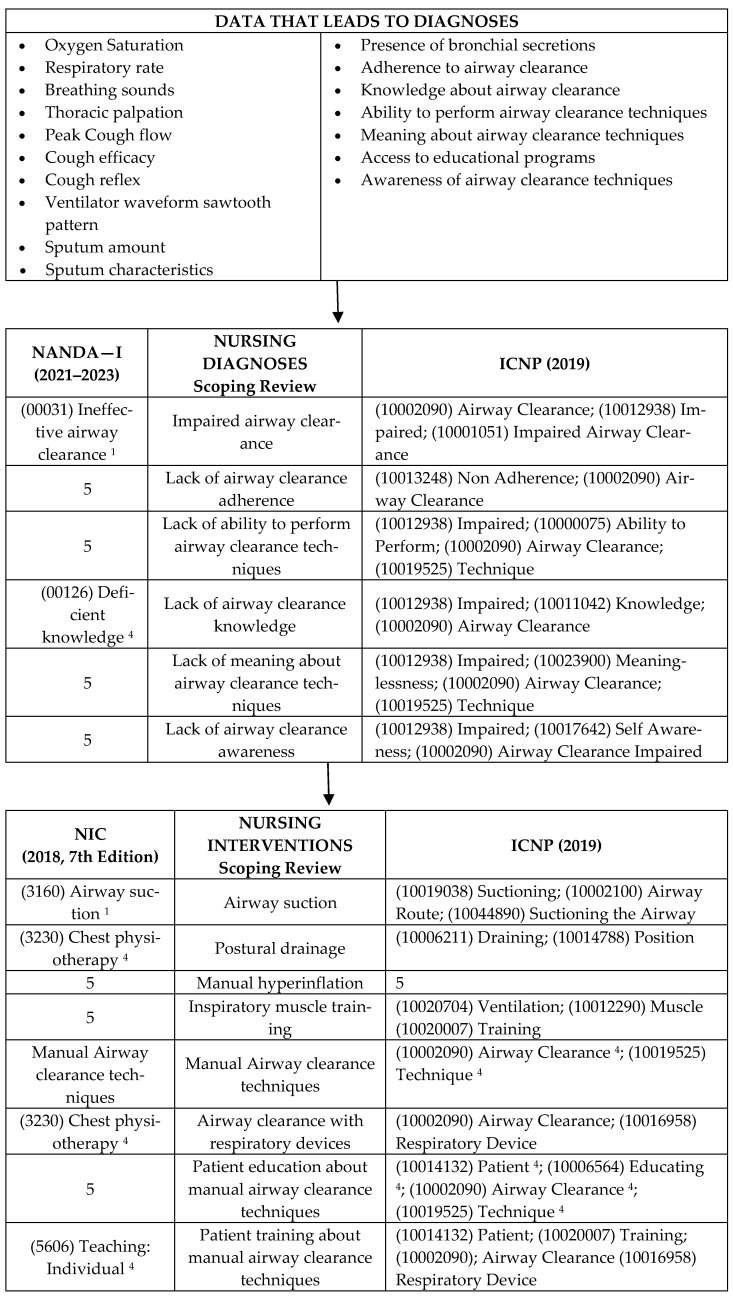

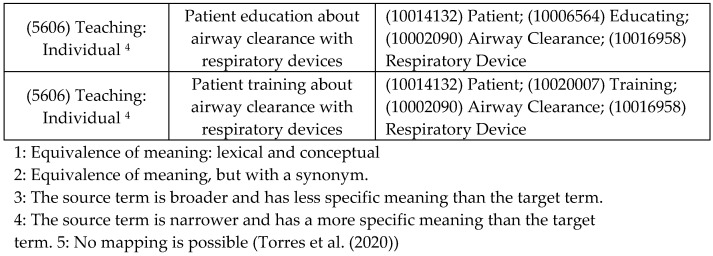
Summary of the results [137].

To resume the information in this review, Figure 2 presents a schematic summary of the results.

## 4. Discussion

The current scoping review is the first to explore the nursing process, addressing the nursing focus “airway clearance”. Thus, the nursing diagnoses, the data that can lead to those diagnoses, and the nursing interventions that positively address nursing diagnoses in the airway clearance domain have been described to answer the review questions.

### 4.1. Clinical Data

According to this review, there are two kinds of clinical data: physiologic data addressing patients’ signs and symptoms and cognitive data related to education strategies.

The retention of bronchial secretions may lead to airway obstruction, increased work of breathing, hypoxia, and respiratory failure. Therefore, the most commonly reported clinical data were related to the presence of retained secretions, such as oxygen saturation (e.g., [21]), sputum amount (e.g., [17]), or breathing sounds (e.g., [25]). These results are present in all contexts of nursing practice, whether in ICU patients (e.g., [127]), hospital conventional wards (e.g., [4]), or community-based care (e.g., [119]).

All of these studies suggest that the most important data that leads to airway clearance impairment is oxygen desaturation, increasing sputum, and the presence of rhonchi during auscultation. Furthermore, we found other data related to cough, as its presence and efficacy are essential for bronchial clearance. Data such as the cough reflex (e.g., [42]), peak cough flow (e.g., [32]), and cough efficacy (e.g., [34]) were found in this review. In a Cochrane systematic review, Morrow et al. (2013) reported that patients with neuromuscular diseases are more likely to have acute respiratory infections due to an ineffective cough related to a lack of muscular tonus [86]. In 2017, Auger et al., in a systematic review, concluded that a peak expiratory flow below 160 L/min indicates a high risk of ineffective cough [38]. Although not directly linked to airway clearance, these data are highly relevant to nursing diagnoses.

The literature reviewed in this study suggests that patients with airway clearance impairment must adapt to physical and psychological changes. This requires the processing of much information concerning airway clearance, demanding new skills. These patients experience a health/illness transition, making it necessary to integrate new knowledge and new skills to lead to behavioral change and achieve a healthy transition [138]. Empowerment and engagement are key parts of this process, starting with awareness and evolving to the improvement of knowledge and the ability to optimize decision-making, leading to a healthy transition [138]. The most common data in this dimension were related to knowledge and ability to perform airway clearance techniques (e.g., [44]), adherence to airway clearance (e.g., [20]), and meaning about airway clearance techniques (e.g., [27]).

A study by Sherman et al. (2019) highlighted a critical gap regarding adherence to airway clearance techniques over time [20]. The results showed that the adherence rate decreased over time due to the patients’ lack of confidence in their ability to perform airway clearance techniques, the perception that self-management of bronchial secretions would not be adequate, difficulties in integrating airway clearance techniques into their daily routine, and the feeling that self-performed airway cleaning techniques would not be necessary.

Hester et al. (2018), analyzing educational programs in patients with bronchiectasis, concluded that more information and better guidance in self-management skills are needed despite the clear potential for such interventions to produce tangible patient benefits [27]. These results highlight the need for a patient-centered approach rather than standardized educational programs with no apparent relation to the patient’s needs. These data are vital for better adapting educational programs to patient needs and for better understanding the causes of lack of treatment adherence. According to these authors, education should be addressed with a patient-centered approach that incorporates knowledge and self-management skills.

### 4.2. Nursing Diagnoses

Regarding the nursing diagnoses, the more frequent were mainly based on signs and symptom management. This diagnostic is already present in ICNP^®^, described as “Impaired Airway Clearance” (ICNP^®^) [1] or in NANDA International, Inc., as “Ineffective airway clearance” [139].

However, nursing care is focused on more than physiologic function impairment and signs and symptoms management. In addition to “Impaired airway clearance”, other nursing diagnoses were identified, for example, related to the ability or adherence to performing airway clearance, which may alone or together contribute to airway clearance impairment. These diagnoses are linked to another dimension of nursing care related to education strategies and are vital in the client-centered care model. The main goal is to develop the patient’s cognitive, behavioral, and emotional skills, expecting improved performance and hoping to obtain mastery to deal with new situations and facilitate the transition process [138]. The transition has a beginning and an end; it begins with awareness of the change and ends fluidly when the person assumes the new roles and develops the necessary skills to achieve a feeling of well-being or the desired quality of life [138]. This fact leads to the incorporation of life changes to better adapt to new conditions. In this dimension, we seek to promote self-management skills, moving from a model centered on professional knowledge to a collaborative model focused on patients’ needs and decisions.

In a systematic review, Schrijver et al. (2023) highlighted the importance of self-management skills in COPD patients to successfully manage the disease and the associated emotional and practical issues. Moreover, the study concluded that self-management is associated with improved health-related quality of life and a decreased probability of respiratory-related hospital admissions [119]. Considering the review results, this seems to be a relevant nursing diagnosis in the “airway clearance” domain.

Nurses recognize the patient’s potential to adapt to the new condition, leading to the development of response patterns that express the presence of risks or reveal signs of a healthy transition. Nursing diagnoses such as “Lack of awareness about airway clearance techniques” (e.g., [20]), “Lack of airway clearance adherence” (e.g., [29]), or “Lack of airway clearance meaning” (e.g., [29]) are essential for developing patient cognitive, behavioral, and emotional skills, leading to mastery being particularly relevant to nursing as it facilitates the transition process.

Of the six nursing diagnoses identified in the scoping review, one can be mapped to NANDA-I by “equivalence of meaning”. The six diagnoses are fully mapped with ICNP.

### 4.3. Nursing Interventions

Finally, the reviewed literature pinpointed some nursing interventions that positively address nursing diagnoses in the airway clearance domain. The majority of nursing interventions found were related to signs and symptom management, such as airway suction (e.g., [19]), postural drainage (e.g., [122]), manual airway clearance techniques (e.g., [126]), or airway clearance with mechanical devices (e.g., [125]).

Diverse techniques, devices, and nursing interventions aimed at performing airway clearance in any context of clinical practice are currently available. However, its multiplicity raises a pertinent question about choosing one technique or procedure over another.

Airway suction is mainly used for patients who are unable to cough or expel bronchial secretions, either because of unconscious cognitive problems, neuromuscular disease, or other reasons. Postural drainage and manual airway clearance techniques, such as chest percussion, chest vibration, or the active cycle of breathing technique, are also essential nursing interventions for reducing airway clearance impairment. The most commonly used airway clearance device is the mechanical insufflator/exsufflator. Several studies have confirmed that it increases cough effectiveness by increasing peak cough flow, particularly in patients with neuromuscular diseases [127]. Other mechanical devices frequently used are oscillatory and high-frequency chest-wall oscillators [121].

To date, there is not enough evidence to support the superiority of one technique, procedure, or device over another in different contexts of clinical practice [23].

From the patient’s perspective, the main goal is to combine the best effect with the lowest possible incidence of side effects and adverse events. The overall effectiveness of these treatments is influenced by several patient-related factors. Treatment adherence is fundamental and largely depends on satisfaction, motivation, and perceived effectiveness. Therefore, patients should be involved in choosing the airway cleaning technique or device that best meets their needs, considering variables such as comfort, convenience, flexibility, practicality, and cost, among others. No articles on this subject were found in this review.

Educational interventions are particularly relevant for diminishing airway clearance impairment complications [119]. Increased knowledge and skills empower individuals to be more involved in their healthcare and to participate in shared decision-making. Lee et al. (2023) concluded that education interventions increase knowledge, ability, and self-efficacy, improve health-related quality of life, and reduce hospitalization in patients with pulmonary fibrosis [140].

Although educational interventions appear to be particularly relevant to diminishing airway clearance impairment, we need more information regarding patient-tailored education programs. All the studies in this review reported predesigned educational programs rather than patient-tailored programs. This fact is particularly relevant because awareness, engagement, knowledge, and ability are critical factors in nursing practice. This fact is significant because it is complicated to evaluate the efficiency of these programs if we do not isolate these variables.

All ten nursing interventions can be mapped to the NIC, but only one by “equivalence meaning”; all the rest are specifications of more comprehensive NIC interventions. Like in nursing diagnoses, ICNP allows us to map practically all interventions, with three of the nursing interventions that emerged from the review being at a more specific level.

In summary, it is evident that multiaxial classifications that provide “building blocks” have more significant potential to formally represent nursing care.

### 4.4. Strengths and Limitations

A strength of this scoping review is that the evidence found is generalizable to other contexts, considering that studies from different regions worldwide were included. On the other hand, specific limitations were inherent to a scoping review: the amount of data generated and the absence of synthesis, as a scoping review does not synthesize. Furthermore, a systematic evaluation of the quality of the articles included in this review was not carried out. This option was based on the inclusive nature of the review, as it would be essential to provide a comprehensive view of the topic of the study. This study did not include gray literature, as the authors have opted for peer-reviewed articles.

## 5. Conclusions

The findings of this review provide a vital contribution to systematizing the nursing process related to the nursing focus “airway clearance”, highlighting the diagnostic hypotheses that derive from this focus, the relevant clinical data that can lead to those diagnoses, and nursing interventions that positively address the nursing diagnoses identified. The results of this review may improve nursing decisions, contributing to improving nursing care quality.

Future studies should consider the need for consensus on the nursing process related to the focus on “airway clearance”, since that is not the aim of a scoping review. For example, by using the Delphi method, it would be important to evaluate whether an extended group of experts agrees with these review findings. This could potentially create an opportunity for reflection and eventually generate more data, diagnoses, and/or nursing interventions.

### Implications for Practice

By mapping the literature regarding data that can lead to nursing diagnoses related to airway clearance, it makes it possible to summarize those data, creating a dataset available for nurses to use in clinical practice. This fact is particularly relevant because it helps to identify diagnoses more clearly by allowing different nurses to use the same data to arrive at the same diagnoses, increasing the comparability of nursing care. Furthermore, this dataset can allow nurses to improve their diagnostic process in a more systematized way and based on the most current scientific evidence.

This scoping review also identified nursing interventions validated, through clinical studies, to be effective in improving nursing diagnoses in the air clearance domain. Therefore, by summarizing that information, it was possible to identify a set of evidence-based nursing interventions that nurses can use in their clinical practice to address diagnoses in this domain. A high level of uniformity is necessary when collecting information to enable its later use, in particular regarding the production of outcomes that can reveal the contribution of nursing care to the population’s health. Therefore, this review may contribute to the improvement of nursing information systems regarding the production of reliable nursing outcomes.

## Figures and Tables

**Table 1:** Clinical data, diagnoses, and nursing interventions as described in the included studies.

Authors	Study Design	Clinical Data	Diagnoses	Interventions
Templeman et al. (2020) Scotland [16]	Systematic review	Cough efficacy Cough reflex		Inspiratory muscle training
Westerdahl et al. (2019) UK [17]	Cross-sectional study	Sputum Amount	Impaired airway clearance	Manual ACT Airway clearance with respiratory devices
Jaiswal and Das et al. (2019) India [18]	RCT		Impaired airway clearance	Manual ACT Airway clearance with respiratory devices Patient education about manual ACT Patient training about manual ACT Patient education about airway clearance with respiratory devices Patient training about airway clearance with respiratory devices
Shamali et al. (2019) Australia [19]	RCT	Presence of bronchial secretions	Impaired airway clearance	Airway suction
Sherman et al. (2019) UK [20]	Observational study	Adherence to airway clearance Awareness of ACT	Lack of airway clearance adherence	
Yazdannik et al. (2019) Iran [21]	Cross-sectional study	Oxygen Saturation	Impaired airway clearance	Airway suction
Tomar et al. (2019) India [22]	RCT	Presence of bronchial secretions	Impaired airway clearance	Manual ACT
Wilson et al. (2019) USA [23]	Systematic review	Presence of bronchial secretions	Impaired airway clearance	Airway clearance with respiratory devices Manual ACT Patient education about manual ACT Patient training about manual ACT Patient education about airway clearance with respiratory devices Patient training about airway clearance with respiratory devices
Allen et al. (2018) UK [24]	Case study	Peak cough flow	Impaired airway clearance	Airway clearance with respiratory devices
Ferreira de Camillis et al. (2018) Brasil [25]	RCT	Sputum amount Breathing sounds Oxygen Saturation Respiratory rate	Impaired airway clearance	Postural drainage Airway clearance with respiratory devices Manual ACT
Pozuelo-Carrascosa et al. (2018) Chile [26]	Systematic review	Oxygen Saturation Cough Efficacy Cough reflex	Impaired airway clearance	Manual ACT Postural drainage
Hester et al. (2018) UK [27]	Qualitative study	Access to educational programs Ability to perform ACT Knowledge about airway clearance Meaning about ACT	Lack of airway clearance knowledge Lack of airway clearance adherence	Patient education about manual ACT Patient training about manual ACT Promoting care plan
Hill et al. (2018) UK [28]	Systematic review		Impaired airway clearance	Patient education about manual ACT Inspiratory muscle training
Kelly et al. (2018) UK [29]	Systematic review	Adherence to airway clearance Ability to perform ACT Knowledge about airway clearance	Impaired airway clearance Lack of ability to perform ACT Lack of airway clearance knowledge	Promoting care plan Patient education about manual ACT Patient training about manual ACT
Ghaleb et al. (2017) Saudi Arabia [30]	Cross-sectional study		Impaired airway clearance	Airway suction
Spapen et al. (2017) Belgium [31]	Systematic review	Oxygen Saturation Sputum Amount	Impaired airway clearance	Manual ACT
McIlwaine et al. (2017) UK [32]	Expert panel	Peak cough flow	Impaired airway clearance Lack of airway clearance knowledge	Manual ACT Airway clearance with respiratory devices Postural drainage Patient education about manual ACT
Gonçalves et al. (2017) Brasil [33]	Systematic review	Breathing sounds Oxygen Saturation Respiratory rate Cough efficacy Sputum amount	Impaired airway clearance	Airway suction Manual hyperinflation
Wang et al. (2017) China [34]	Cross-sectional study	Oxygen Saturation Cough efficacy	Impaired airway clearance	
Chuang et al. (2017) Taiwan [35]	RCT	Oxygen Saturation Cough efficacy	Impaired airway clearance	Airway clearance with respiratory devices Manual ACT
D’Abrosca et al. (2017) Italy [36]	Observational study	Sputum amount	Impaired airway clearance Lack of ability to perform ACT Lack of airway clearance knowledge	Airway clearance with respiratory devices Patient education about manual ACT Patient education about airway clearance with respiratory devices Patient training about manual ACT Patient training about airway clearance with respiratory devices
Morrison et al. (2017) UK [37]	Systematic review		Impaired airway clearance	Airway clearance with respiratory devices Manual ACT
Auger et al. (2017) France [38]	Systematic review	Peak cough flow Sputum amount	Impaired airway clearance	Airway clearance with respiratory devices Manual ACT
Borges et al. (2017) Brasil [39]	Systematic review		Impaired airway clearance	Manual ACT
McCormack et al. (2017) USA [40]	Systematic review	Sputum amount	Impaired airway clearance Lack of ability to perform ACT Lack of knowledge about ACT	Airway clearance with respiratory devices Manual ACT Patient education about manual ACT Patient education about airway clearance with respiratory devices Patient training about manual ACT Patient training about airway clearance with respiratory devices
Rodriguez Hortal et al. (2017) Sweden [41]	RCT		Impaired airway clearance	Airway clearance with respiratory devices
Rose et al. (2017) USA [42]	Systematic review	Presence of bronchial secretions Cough efficacy Cough reflex Peak cough flow	Impaired airway clearance	Manual ACT Airway clearance with respiratory devices
Torres-Sanchez et al. (2017) Spain [43]	Systematic review		Impaired airway clearance	Manual ACT
Boulet (2016) Canada [44]	Expert panel	Ability to perform ACT Knowledge about airway clearance	Lack of ability to perform ACT Lack of knowledge about ACT Lack of meaning about ACT	Promoting care plan Patient education about manual ACT Patient training about manual ACT
Berry et al. (2016) UK [45]	Observational study	Breathing sounds Thoracic palpation Presence of bronchial secretions Ventilator waveform sawtooth pattern	Impaired airway clearance	
Arcuri et al. (2016) Brasil [46]	Systematic review	Cough efficacy	Impaired airway clearance	Manual ACT Airway clearance with respiratory devices
Laciuga et al. (2016) USA [47]	Observational study	Cough efficacy Peak cough flow		
Lucchini et al. (2016) USA [48]	Observational study		Impaired airway clearance	Airway suction
Pascoal et al. (2016) Brasil [49]	Cross-sectional study	Presence of bronchial secretions Cough efficacy Respiratory rate Breathing sounds Sputum amount	Impaired airway clearance	
Jena et al. (2016) India [50]	RCT		Impaired airway clearance	Airway suction
Berry et al. (2016) UK [45]	Observational study	Peak cough flow Meaning about ACT	Lack of airway clearance adherence Lack of meaning about ACT	Patient education about manual ACT
Button et al. (2016) Australia [51]	Systematic review	Presence of bronchial secretions	Impaired airway clearance	Manual ACT Airway clearance with respiratory devices Postural drainage Patient education about manual ACT Patient education about airway clearance with respiratory devices
McKoy et al. (2016) USA [52]	Systematic review	Sputum amount Breathing sounds	Impaired airway clearance	Manual ACT Airway clearance with respiratory devices Postural drainage Patient education about manual ACT Patient education about airway clearance with respiratory devices
Dwyer et al. (2015) Australia [53]	RCT		Impaired airway clearance	Manual ACT Airway clearance with respiratory devices
Gastaldi et al. (2015) Brasil [54]	Cross-sectional study		Impaired airway clearance	Airway clearance with respiratory devices
Liu et al. (2015) China [55]	Quasi-experimental study	Oxygen saturation Respiratory rate Breathing sounds Presence of bronchial secretions	Impaired airway clearance	Airway suction
Ozden et al. (2015) Turkey [56]	Quasi-experimental study	Presence of bronchial secretions Oxygen saturation Cough efficacy Breathing sounds Ventilator waveform sawtooth pattern Sputum amount Respiratory rate	Impaired airway clearance	Airway suction
Sole et al. (2015) USA [57]	Descriptive Study	Presence of bronchial secretions Oxygen saturation Cough efficacy Breathing sounds Ventilator waveform sawtooth pattern Sputum amount	Impaired airway clearance	Airway suction
Reychler et al. (2015) Brasil [58]	Observational study	Ability to perform ACT Adherence to airway clearance	Lack of airway clearance adherence Lack of ability to perform ACT	Airway clearance with respiratory devices Patient education about airway clearance with respiratory devices Patient training about airway clearance with respiratory devices
Lee et al. (2015) USA [59]	Systematic review		Impaired airway clearance	Manual ACT Airway clearance with respiratory devices
Lee and Park et al. (2015) Australia [60]	Systematic review	Sputum amount	Impaired airway clearance	Manual ACT Airway clearance with respiratory devices
McCullough et al. (2014) USA [61]	Systematic review	Adherence to airway clearance	Lack of airway clearance adherence	Patient education about manual ACT Patient training about manual ACT
Morgan et al.. (2016) Canada [62]	Systematic review	Sputum amount	Impaired airway clearance	Manual ACT
Anand (2014) India [63]	Comparative Study	Sputum amount Presence of bronchial secretions	Impaired airway clearance	Manual ACT Patient education about manual ACT Patient training about manual ACT
Caparros (2014) USA [64]	Systematic review	Oxygen Saturation Presence of bronchial secretions	Impaired airway clearance	Airway suction
Cork et al. (2014) Australia [65]	Case Study	Presence of bronchial secretions	Impaired airway clearance	Airway suction Manual hyperinflation Manual ACT Postural drainage
Dos Santos et al. (2014) Brasil [66]	Quasi-experimental study	Oxygen Saturation	Impaired airway clearance	Manual hyperinflation Manual ACT
Esguerra-Gonzales et al. (2014) USA [67]	Cross-sectional study	Adherence to airway clearance	Impaired airway clearance Lack of airway clearance adherence	Manual ACT Airway clearance with respiratory devices
Guimarães et al. (2014) Brasil [68]	RCT	Presence of bronchial secretions Knowledge about airway clearance Ability to perform ACT	Impaired airway clearance Lack of ability to perform ACT Lack of airway clearance knowledge	Manual ACT Airway clearance with respiratory devices Patient education about manual ACT Patient education about airway clearance with respiratory devices
Kohan et al. (2014) Iran [69]	RCT	Cough efficacy	Impaired airway clearance	Manual ACT Airway suction
O’Donohoe and Fullen et al. (2014) UK [70]	Systematic review	Adherence to airway clearance Meaning about ACT	Lack of airway clearance adherence Lack of meaning about ACT Lack of airway clearance knowledge	
Ntoumenopoulos et al. (2014) Australia [71]	Observational study	Breathing sounds Thoracic palpation Cough reflex Oxygen saturation Peak cough flow	Impaired airway clearance	Manual hyperinflation Manual ACT
Zanni et al. (2014) UK [72]	Quasi-experimental study	Adherence to airway clearance Awareness of ACT Knowledge about airway clearance Ability to perform ACT	Lack of airway clearance adherence Lack of ability to perform ACT Lack of airway clearance knowledge Lack of awareness about ACT	Patient education about manual ACT Patient training about manual ACT
Shukla et al. (2014) India [73]	Observational study	Sputum amount	Impaired airway clearance	Manual ACT Airway clearance with respiratory devices
Standford et al. (2014) UK [74]	Observational study		Impaired airway clearance	Manual ACT
Torres-Castro et al. (2014) Chile [75]	Cross-sectional study	Peak cough flow Cough efficacy	Impaired airway clearance	Manual ACT
Savage (2014) USA [76]	Systematic review	Knowledge about airway clearance Adherence to airway clearance	Lack of airway clearance adherence Lack of airway clearance knowledge	Patient education about manual ACT Patient training about manual ACT
Zwerink et al. (2014) Australia [77]	Systematic review	Ability to perform ACT	Lack of airway clearance adherence Lack of ability to perform ACT	Promoting care plan Patient education about manual ACT Patient training about manual ACT
Flores et al. (2013) UK [78]	Cross-sectional study	Adherence to airway clearance Meaning about ACT	Lack of airway clearance adherence	Patient education about manual ACT Patient training about manual ACT
Esguerra-Gonzalez et al. (2013) USA [79]	Experimental Study		Impaired airway clearance	Manual ACT Airway clearance with respiratory devices
Yang (2013) China [80]	Systematic review		Impaired airway clearance	Manual ACT Airway clearance with respiratory devices
Volsky (2013) UK [81]	Systematic review	Presence of bronchial secretions Cough reflex Cough efficacy Adherence to airway clearance	Impaired airway clearance Lack of airway clearance adherence	Manual ACT Airway clearance with respiratory devices Postural drainage Patient education about manual ACT Patient training about manual ACT Patient education about airway clearance with respiratory devices Patient training about airway clearance with respiratory devices
Maggiore et al. (2013) Italy [82]	Observational study	Breathing sounds Oxygen Saturation Presence of bronchial secretions	Impaired airway clearance	Manual Hyperinflation Airway suction
Nicolini et al. (2013) Italy [83]	RCT	Presence of bronchial secretions	Impaired airway clearance	Manual ACT Airway clearance with respiratory devices
Warnock et al. (2015) USA [84]	Systematic review		Impaired airway clearance	Manual ACT Airway clearance with respiratory devices
Andrews et al. (2013) USA [85]	Systematic review	Cough reflex Peak cough flow Oxygen Saturation Breathing Sounds Presence of bronchial secretions Sputum amount Cough efficacy	Impaired airway clearance	Manual ACT Airway clearance with respiratory devices Postural drainage
Morrow et al. (2013) Republica of South Africa) [86]	Systematic review	Peak cough flow Cough efficacy	Impaired airway clearance	Airway clearance with respiratory devices
Strickland et al. (2013) USA [87]	Systematic review	Peak cough flow Cough efficacy Oxygen Saturation Presence of bronchial secretions Knowledge about airway clearance Ability to perform ACT	Impaired airway clearance Lack of ability to perform ACT Lack of airway clearance knowledge	Manual ACT Airway clearance with respiratory devices Patient education about manual ACT Patient training about manual ACT Patient education about airway clearance with respiratory devices Patient training about airway clearance with respiratory devices
Clinkscale et al. (2012) USA [88]	RCT	Presence of bronchial secretions Knowledge about airway clearance Ability to perform ACT	Impaired airway clearance Lack of ability to perform ACT Lack of airway clearance knowledge	Patient education about manual ACT Patient training about manual ACT
Corley et al. (2012) Australia [89]	RCT	Oxygen Saturation Sputum amount	Impaired airway clearance	Airway suction
Cross et al. (2012) UK [90]	RCT	Oxygen Saturation Sputum amount Breathing sounds Knowledge about airway clearance Ability to perform ACT	Impaired airway clearance Lack of ability to perform ACT Lack of airway clearance knowledge	Manual ACT Patient education about manual ACT
Figueiredo et al. (2012) Brazil [91]	RCT	Presence of bronchial secretions Knowledge about airway clearance Ability to perform ACT	Impaired airway clearance Lack of ability to perform ACT Lack of airway clearance knowledge	Manual ACT Airway clearance with respiratory devices Patient education about manual ACT Patient training about manual ACT Patient education about airway clearance with respiratory devices Patient training about airway clearance with respiratory devices
Ozden and Gorgulu (2015) Turkey) [56]	Observational study	Oxygen Saturation Presence of bronchial secretions	Impaired airway clearance	Airway suction
Park et al. (2012) South Korea [92]	RCT	Sputum amount Oxygen Saturation	Impaired airway clearance	Manual ACT Airway clearance with respiratory devices
Lewis and Olds (2012) Australia [93]	Systematic review		Impaired airway clearance	Manual ACT Manual hyperinflation
Osadnik et al. (2012) Australia [94]	Systematic review		Impaired airway clearance	Manual ACT Airway clearance with respiratory devices
Paulus et al. (2012) Netherland [95]	Systematic review	Presence of bronchial secretions Cough reflex Oxygen saturation	Impaired airway clearance	Manual hyperinflation Airway suction Manual ACT
Naue et al. (2011) Brazil [96]	RCT	Presence of bronchial secretions	Impaired airway clearance	Manual hyperinflation Manual ACT Airway suction
Lavery et al. (2011) UK [97]	RCT	Knowledge about airway clearance Meaning about ACT	Lack of airway clearance adherence Lack of airway clearance knowledge	Patient education about manual ACT Patient training about manual ACT
Mahajan et al. (2011) USA [98]	RCT	Sputum amount	Impaired airway clearance	Airway clearance with respiratory devices
Suh et al. (2011) South Korea [99]	Experimental Study	Oxygen saturation	Impaired airway clearance	Airway suction Manual ACT
Aggarwal et al. (2010) India [100]	RCT	Oxygen saturation Respiratory rate Peak cough flow Knowledge about airway clearance Ability to perform ACT	Impaired airway clearance Lack of ability to perform ACT Lack of airway clearance knowledge	Manual ACT Airway clearance with respiratory devices Patient education about manual ACT Patient training about manual ACT Patient education about airway clearance with respiratory devices Patient training about airway clearance with respiratory devices
Davidson et al. (2010) Brazil [101]	Case Study	Sputum characteristics	Impaired airway clearance	Postural drainage
Mattos de Castro et al. (2010) Brazil [102]	RCT	Sputum amount Presence of bronchial secretions Breathing sounds	Impaired airway clearance	Manual ACT
Pattanshetty et al. (2010) India [103]	RCT		Impaired airway clearance	Manual ACT Manual Hyperinflation Airway suction Postural drainage
Kempainen et al. (2010) USA [104]	RCT	Sputum amount Cough efficacy	Impaired airway clearance	Airway clearance with respiratory devices
Kjonegaard et al. (2010) USA [105]	Comparative study	Oxygen saturation Sputum amount	Impaired airway clearance	Airway suction Manual hyperinflation
Naraparaju et al. (2010) India [106]	RCT	Sputum amount Ability to perform ACT Knowledge about airway clearance	Impaired airway clearance	Manual ACT Airway clearance with respiratory devices Patient education about manual ACT Patient training about manual ACT Patient education about airway clearance with respiratory devices Patient training about airway clearance with respiratory devices
Osman et al. (2010) UK [107]	RCT	Oxygen Saturation Sputum amount	Impaired airway clearance	Manual ACT Airway clearance with respiratory devices Postural drainage Patient education about manual ACT
Wang et al. (2010) China [108]	RCT		Impaired airway clearance	Airway clearance with respiratory devices Patient education about manual ACT Patient education about airway clearance with respiratory devices
Saowanee et al. (2010) Thailand [109]	Multicase Study	Peak cough flow Ability to perform ACT	Impaired airway clearance Lack of ability to perform ACT	Manual ACT Patient education about manual ACT
Hill et al. (2010) Australia [110]	Systematic review	Oxygen saturation	Impaired airway clearance	Manual ACT Airway clearance with respiratory devices
Nowobilski et al. (2010) Poland [111]	Systematic review	Oxygen Saturation	Impaired airway clearance	Manual ACT Airway clearance with respiratory devices Postural drainage Patient education about manual ACT
Darlene Reid et al. (2010) Canada [112]	Systematic review	Cough efficacy	Impaired airway clearance	Manual ACT Airway clearance with respiratory devices
Allen and O’Leary (2018) USA [113]	Quasi-experimental study	Oxygen Saturation Presence of bronchial secretions	Impaired airway clearance	Manual ACT Airway clearance with respiratory devices
Chatwin and Simonds (2009) UK [114]	RCT	Oxygen Saturation Breathing sounds Cough efficacy Sputum amount Presence of bronchial secretions Peak cough flow Respiratory rate		Manual ACT Airway clearance with respiratory devices
Pedersen et al. (2009) Danmark [115]	Systematic review	Cough reflex Cough efficacy Presence of bronchial secretions Oxygen Saturation Breathing sounds	Impaired airway clearance	Airway suction
Toussaint (2009) Belgium [116]	Cross-sectional study	Cough efficacy Peak cough flow	Impaired airway clearance	Manual ACT
Lavery et al. (2007) UK [117]	Focus group study	Adherence to airway clearance Knowledge about airway clearance Ability to perform ACT Meaning about ACT	Lack of meaning about ACT Lack of ability to perform ACT Lack of airway clearance knowledge	Patient education about manual ACT Patient training about manual ACT
Kaneko H. et al. (2022) Japan [118]	RCT	Cough efficacy Peak cough flow	Impaired airway clearance	Inspiratory muscle training
Schrijver J. et al. (2022) Netherland [119]	Systematic review		Impaired airway clearance Lack of airway clearance knowledge	Promoting care plan Patient education about manual ACT Patient training about manual ACT
Zisi D et al. (2022) Greece [120]	Systematic review	Sputum amount Presence of bronchial secretions Peak cough flow	Impaired airway clearance	Manual ACT Patient education about manual ACT
Alghamdi S.M. et al. (2023) UK [121]	RCT	Sputum amount Peak cough flow	Impaired airway clearance	Airway clearance with respiratory devices Patient education about manual ACT Patient training about manual ACT
Chandrasekar S et al. (2022) India [122]	RCT	Sputum amount Ability to perform ACT Knowledge about airway clearance	Impaired airway clearance Lack of ability to perform ACT Lack of airway clearance knowledge	Manual ACT Postural drainage Airway clearance with respiratory devices Patient education about manual ACT Patient training about manual ACT Patient education about Airway clearance with respiratory devices Patient training about Airway clearance with respiratory devices
Zhong, J. et al. (2022) China [123]	China	Sputum amount Presence of bronchial secretions Sputum characteristics oxygen saturation Ability to perform ACT	Impaired airway clearance Lack of ability to perform ACT	Manual ACT Patient education about manual ACT Patient training about manual ACT
Apps, C. et al. (2022) UK [124]	Case study report	Presence of bronchial secretions Cough efficacy Breathing sounds Cough reflex	Impaired airway clearance	Airway suction Postural drainage Airway clearance with respiratory devices Manual ACT
Mitropoulou, G. et al. (2023) Switzerland [125]	Observational study	Presence of bronchial secretions Cough efficacy Peak cough flow	Impaired airway clearance Lack of ability to perform ACT	Airway clearance with respiratory devices Manual ACT Patient education about manual ACT Patient education about airway clearance with respiratory devices Manual ACT
Chen, X. et al. (2022) China [126]	Systematic review	oxygen saturation Sputum amount Breathing sounds	Impaired airway clearance	Manual ACT Postural drainage Airway clearance with respiratory devices Patient education about manual ACT Patient education about airway clearance with respiratory devices
Swingwood, E. et al. (2022) UK [127]	Scoping review	Presence of bronchial secretions Oxygen saturation Breathing sounds cough reflex Cough efficacy Peak cough flow	Impaired airway clearance	Airway clearance with respiratory devices
AbdelHalim, H. et al. (2016) Egypt [128]	RCT	Presence of bronchial secretions Oxygen saturation Breathing sounds	Impaired airway clearance	Manual ACT Postural drainage
Main, E et al. (2023) USA [129]	Systematic review	Presence of bronchial secretions Sputum characteristics Sputum amount	Impaired airway clearance	Manual ACT Airway clearance with respiratory devices
Hegland et al. (2014) USA [130]	Observational Study	Cough efficacy Peak cough flow		Manual ACT
Goni-Viguria, R. et al. (2017) Spain [131]	Narrative review	Presence of bronchial secretions Oxygen saturation Cough efficacy	Impaired airway clearance	Manual ACT Postural drainage Airway clearance with respiratory devices
Ward, N et al. (2018) Australia [132]	Cross-sectional Study		Impaired airway clearance	Manual ACT Postural drainage Airway clearance with respiratory devices
Mcllwaine, M. et al. (2013) USA [133]	RCT	Presence of bronchial secretions Oxygen saturation Breathing sounds	Impaired airway clearance	Manual ACT Airway clearance with respiratory devices
Varekojis, S. et al. (2003) USA [134]	Quasi-experimental study	Sputum characteristics Sputum amount	Impaired airway clearance	Manual ACT Postural drainage Airway clearance with respiratory devices
Poncin, W. et al. (2017) Belgium [135]	Quasi-experimental study	Sputum amount	Impaired airway clearance	Autogenic drainage
McCool, D. et al. (2006) USA [136]	Systematic review	Presence of bronchial secretions Oxygen saturation Breathing sounds cough reflex Cough efficacy	Impaired airway clearance	Manual ACT Postural drainage Airway clearance with respiratory devices Inspiratory muscle training

RCT: Randomized controlled trail; ACT: Airway Clearance technique.

## Data Availability

For data supporting reported results, please contact the authors of this review.

## Supplementary Materials

The following supporting information can be downloaded at: https://www.mdpi.com/article/10.3390/nursrep14030140/s1, Table S1: Searching strategy.

## Public Involvement Statement

There is no public involvement in any aspect of this research.

## Guidelines and Standards Statement

This manuscript was drafted against the “The PRISMA2020 statement: An updated guideline for reporting systematic reviews” for systematic review research.
