# Supplementary material for: Nursing Process Related to the Nursing Focus “Airway Clearance”: A Scoping Review
Source: Nurs Rep. 2024 Jul 31;14(3):1871–96. doi: 10.3390/nursrep14030140 (PMC11348224; doi:10.3390/nursrep14030140)
Supplement: Supplementary file 1 [file nursrep-14-00140-s001.zip › nursrep-3094878-supplementary.pdf]

Supplementary Table S1: Search strategy

CINAHL Complete; MedicLatina; MEDLINE (PubMed).

Filter: all adult

Filter: Languages: English; Portuguese and Spanish

Search conducted in March 19th 2024

Query	Records
TI ((MH "Intermittent Positive Pressure Breathing/AE/CT/ES/EV/MT/NU/ST/UT") NOT (MH "Intermittent Positive Pressure Ventilation/AE/CT/ES/EV/MT/NU/ST/UT") OR (MH "Ineffective Airway Clearance ") OR (MH "Mucociliary Clearance/AE/CT/ES/EV/MT/NU/ST/UT") OR (MH "Work of Breathing/EV/NU") OR (MH "Airway Insertion and Stabilization (Iowa NIC)") OR (MH "Airway Clearance Impairment (Saba CCC)") OR (MH "Airway Suctioning (Iowa NIC)") OR (MH "Suctioning, Nasopharyngeal") OR (MH "Suctioning, Endotracheal") OR (MH "Respiratory Monitoring (Iowa NIC)") OR (MH "Respiratory Management (Iowa NIC)") OR (MH "Nebulizers /EV/NU/ST/SD/UT") OR (MH "Chest Physiotherapy (Iowa NIC)") OR (MH "Cough/CL/CO/DI/NU/RH/RF/SS/TH")) OR AB ((MH "Intermittent Positive Pressure Breathing/AE/CT/ES/EV/MT/NU/ST/UT") NOT (MH "Intermittent Positive Pressure Ventilation/AE/CT/ES/EV/MT/NU/ST/UT") OR (MH "Ineffective Airway Clearance ") OR (MH "Mucociliary Clearance/AE/CT/ES/EV/MT/NU/ST/UT") OR (MH "Work of Breathing/EV/NU") OR (MH "Airway Insertion and Stabilization (Iowa NIC)") OR (MH "Airway Clearance Impairment (Saba CCC)") OR (MH "Airway Suctioning (Iowa NIC)") OR (MH "Suctioning, Nasopharyngeal") OR (MH "Suctioning,	1793

Endotracheal") OR (MH "Respiratory Monitoring (Iowa NIC)") OR (MH "Respiratory Management (Iowa NIC)") OR (MH "Nebulizers /EV/NU/ST/SD/UT") OR (MH "Chest Physiotherapy (Iowa NIC)") OR (MH "Cough/CL/CO/DI/NU/RH/RF/SS/TH")) OR SU ((MH "Intermittent Positive Pressure Breathing/AE/CT/ES/EV/MT/NU/ST/UT") OR (MH "Intermittent Positive Pressure Ventilation/AE/CT/ES/EV/MT/NU/ST/UT") OR (MH "Ineffective Airway Clearance ") OR (MH "Mucociliary Clearance/AE/CT/ES/EV/MT/NU/ST/UT") OR (MH "Work of Breathing/EV/NU") OR (MH "Airway Insertion and Stabilization (Iowa NIC)") OR (MH "Airway Clearance Impairment (Saba CCC)") OR (MH "Airway Suctioning (Iowa NIC)") OR (MH "Suctioning, Nasopharyngeal") OR (MH "Suctioning, Endotracheal") OR (MH "Respiratory Monitoring (Iowa NIC)") OR (MH "Respiratory Management (Iowa NIC)") OR (MH "Nebulizers /EV/NU/ST/SD/UT") OR (MH "Chest Physiotherapy (Iowa NIC)") OR (MH "Cough/CL/CO/DI/NU/RH/RF/SS/TH")) OR (TI postural drainage OR TI mechanical insufflation/exsufflation OR TI active cycle breathing technique OR TI chest wall compression)	

PEDro Physiotherapy Data Base

Filter: all adult

Filter: Languages: English; Portuguese and Spanish

Search conducted in March 19th 2024

Query	Records
"Ineffective Airway Clearance" AND "Mucociliary Clearance" AND "Airway Clearance Impairment" AND "Chest Physiotherapy "	61
